# Aggravation of symptom severity in adult attention-deficit/hyperactivity disorder by latent *Toxoplasma gondii* infection: a case–control study

**DOI:** 10.1038/s41598-020-71084-w

**Published:** 2020-09-01

**Authors:** Alexandra P. Lam, Dominik de Sordi, Helge H. O. Müller, Martin C. Lam, Angelika Carl, Klaus P. Kohse, Alexandra Philipsen

**Affiliations:** 1 Department of Psychiatry and Psychotherapy, University of Bonn, Bonn, Germany; 2 School of Medicine and Health Sciences, Medical Campus University of Oldenburg, Oldenburg, Germany; 3 Division of Epidemiology and Biometry, School of Medicine and Health Sciences, Carl von Ossietzky University of Oldenburg, Oldenburg, Germany; 4 Department of Psychiatry and Psychotherapy, Chair of Integrative Psychiatry and Psychotherapy, Witten/Herdecke University, Gemeinschaftskrankenhaus Herdecke, Witten/Herdecke, Germany; 5 Institute for Laboratory Diagnostics and Microbiology, Klinikum Oldenburg, School of Medicine and Health Sciences, Carl von Ossietzky University of Oldenburg, Oldenburg, Germany

**Keywords:** Parasitic infection, ADHD

## Abstract

*Toxoplasma gondii* (*T. gondii*) has a high worldwide prevalence and an underestimated impact on neuropsychiatric disorders. Previous studies related *T. gondii* to disorders associated with the dysfunctional dopaminergic system. However, an association between *T. gondii* infection and adult attention-deficit/hyperactivity disorder (ADHD) has not yet been studied. In a sex- and age-matched case–control study, we investigated the seropositivity, serointensity, and avidity of latent *T. gondii* infection in adult ADHD patients and examined the influence of those variables on the symptomatology of ADHD. Of 140 participants, 20.0% were seropositive for anti-*T. gondii* IgG and 0% for anti-*T. gondii* IgM. *T. gondii* seropositivity was associated with 2.8-fold increase in the odds of ADHD in a confounder-adjusted multivariable analysis. Age and consumption of raw/undercooked meat were confirmed as significant predictors of *T. gondii* seropositivity. Multiple linear regression analysis of self-rated ADHD-related symptom severity in all participants revealed a significant association with *T. gondii* seropositivity, elevated IgG titers (serointensity), and stronger anti-*T. gondii* IgG avidity. Overall symptom severity was increased in seropositive ADHD patients compared to seronegative subjects with ADHD. In particular, hyperactivity was significantly associated with serointensity. We conclude that there is a high rate of *T. gondii* seropositivity in adults with ADHD. Additionally, our results suggest a clinical impact of latent *T. gondii* infection on ADHD-related symptoms in a serointensity- and avidity-dependent manner.

## Introduction

Adult attention-deficit/hyperactivity disorder (ADHD) is a frequently diagnosed neurodevelopmental disorder with an average worldwide prevalence of 2.8%^1^. This condition is characterized by inattention, impulsivity, and/or hyperactivity^2^. The pathogenesis is considered multifactorial: several genetic and environmental risk factors have been shown to contribute to an increased susceptibility to ADHD, and a wide range of functional and structural brain anomalies, e.g., in the dopaminergic system have been found to be associated with the disorder^3^.

Recent population-based registry studies in children^4–6^ and adults^7–9^ suggest that infections are associated with an increased risk of mental disorders by a direct influence on the nervous system, immune activation, or inflammatory mediators^6,10^. However, studies focusing on the general relationship of infectious agents and ADHD are scarce^11–13^.

*Toxoplasma gondii* (*T. gondii*) is an obligate intracellular parasite with a high (30–50%) worldwide prevalence and growing importance in neuropsychiatric research^14,15^. The parasite shows high affinity for brain tissue, wide distribution in the brain and potential life-long persistence causing latent infections^16,17^. Studies in rodents and humans have already linked *T. gondii* to various neuropsychiatric diseases, especially those associated with a dysfunctional dopaminergic system, e.g., bipolar disorder type I, schizophrenia, and Parkinson’s disease^18,19^. However, the association between *T. gondii* infection and ADHD in adulthood has not yet been investigated.

Today, the most relevant definite host of *T. gondii* is the domestic cat, which harbors the sexual parasitic cycle in the feline intestine and spreads infectious oocysts through feces^20^. The asexual reproduction of the parasite takes place in a broad spectrum of intermediate hosts^21^. While in the past, our ancestors might have been prey of larger cats^21^, humans currently represent dead-end hosts of *T. gondii*^20^. The main risk factors for infection in humans are consuming the parasite’s cysts through cyst-carrying undercooked meat, oocyst-contaminated soil, or contact with fecal material of infected cats^22^. In immunocompetent humans, infections are mostly regarded as clinically asymptomatic^23^. However, the results of animal and behavioral studies showed evidence that latent toxoplasmosis may lead to behavioral changes, the so-called manipulation hypothesis^24–27^. Several studies describe reduced fear displayed by rodents toward cats, which reportedly increases the parasite’s chance of being transmitted to its definite host^15,17^. Studies comparing infected and uninfected humans in terms of personality traits, behavior, and psychomotor performance as well as intelligence suggest the applicability of the manipulation hypothesis to humans^28,29^. In particular, the ability of *T. gondii* to influence and dysregulate dopamine metabolism as well as disease susceptibility genes of its host has come into focus in current research on different psychiatric, neurological, and somatic disorders^16,30–32^. The dopaminergic neurotransmission pathway is also strongly related to ADHD^33,34^. Previous studies discussed that behavioral changes of *T. gondii*-infected rodents might resemble clinical symptoms of patients with ADHD^30^, such as impaired learning capacity and memory^35,36^, rapid loss of concentration^37^, and elevated activity levels^38^. Furthermore, latent toxoplasmosis has been associated with lengthened reaction times^38^, deficits in motor performance^39^, reduced anxiety^40^, and increased novelty-seeking behavior^41^. In concordance, impaired concentration capacity, motor skills and motor control as well as lengthened reaction time^42^, increased novelty-seeking behavior^41^, and an increased risk of injury^43^ have also been described in patients with ADHD.

In a case–control study, we investigated the association between latent *T. gondii* infection and adult ADHD compared to an age- and sex-matched healthy control sample. We further investigated the possible influences of the parasite on the symptomatology of ADHD in detail.

The antigen–antibody binding avidity of immunoglobulin G (IgG) increases over time and provides further information on the age of infection^44^. Furthermore, when linking *T. gondii* with psychiatric diseases, prior research revealed that the robustness of the antibody response (so-called anti-*T. gondii* IgG concentration, *T. gondii* titer, or ‘serointensity’) rather than seropositivity is an important factor that plays a crucial role in behavioral changes^45,46^. However, available data on *T. gondii* seropositivity in ADHD involve only children and do not provide information on serointensity^13,47,48^. In contrast, we included both the anti-*T. gondii* IgG avidity and the measurement of serointensity in this study.

Our main hypothesis is a higher *T. gondii* seropositivity in adult ADHD patients compared to healthy controls. The secondary hypotheses are as follows: we hypothesize that the presence of a latent *T. gondii* infection (seropositivity) and the concentrations of anti-*T. gondii* IgG (serointensity) contribute to the severity of ADHD symptomatology, measured by valid diagnostic instruments.

## Results

### Sample

A total of 193 individuals were contacted for study participation; 41 were ineligible or not interested. Overall, 152 of 193 contacted participants were assessed for eligibility (78.8%). Of these 152, four patients did not want to stop medication for study participation, two participants refused to undergo blood sampling, three patients did not show up for blood sampling for unknown reasons, and two participants handed back incomplete questionnaires. As a consequence, 141 participants [71 subjects with ADHD (50.4%) and 70 healthy controls (49.6%)] were taken into account for analysis. Among 141 individuals screened for IgG and IgM antibodies against *T. gondii*, one participant in the ADHD group (0.7%) had equivocal anti-*T. gondii* IgG titers (serointensity) of 3–6 (U/ml) and was thus excluded from analysis, resulting in a final study sample size of 140 participants.

C-reactive protein (CRP) concentrations below 1 mg/dl were present in 69 (98.6%) individuals in the control group and in 67 patients (95.7%) in the ADHD group. Three participants in the ADHD group (4.3%) had CRP concentrations between 2 and 3 mg/dl. None of the included participants showed any changes in the differential blood count according to acute inflammation or infection. In total, 140 individuals without signs of acute infection were included in the study.

#### Demographic and screening characteristics

The groups were matched for sex and age; thus, the groups were balanced. The sociodemographic characteristics, including medical and family history, of the study population are shown in Table 1. Two controls suffered from specific phobia, such as arachnophobia and claustrophobia, which did not represent exclusion criteria. One male control was suspected to have ADHD in childhood but did not fulfill the criteria of ADHD in adulthood or other psychiatric disorders. Therefore, the participant was further counted in the control group and included in the final analysis. The questionnaire results of both groups are presented in Supplementary Table 2.

**Table 1:** Sample characteristics.

	ADHD (n = 70)	Controls (n = 70)
**Characteristics**		
Sex (male)	36	34
**Age, years**		
Mean (SD)	33 (11)	31 (10)
Range	18–57	18–60
**Body mass index (BMI)**		
Mean (SD)	26 (6.0)	24 (4.2)
Range	17–45	17–35
	**n (%)**	
**Current ADHD medication**	36 (51.4)	0
Methylphenidate	32 (45.7)	0
Atomoxetine hydrochloride, lisdexamfetamine	7 (10.1)	0
**Current other psychopharmacological treatments**	32 (45.7)	7 (10.0)
Antidepressants	19 (27.1)	0
Neuroleptics	6 (8.6)	0
Mood stabilizers	0 (0)	0
Hypnotics, sedatives	3 (4.3)	0
Medication for physical aliments	21 (30.0)	7 (10.0)
**Current comorbid Axis I disorders**	44 (62.9)	3 (4.3)
≥ 1 current clinical disorder	11 (15.7)	1 (1.4)
Affective disorders	31 (44.3)	2 (2.9)
Anxiety disorders	14 (20.0)	2 (2.9)
Eating disorders	3 (4.3)	0
Substance abuse or dependence, current (excludes smoking)	10 (14.3)	0
Substance abuse, lifetime (past and present; excludes smoking)	19 (27.1)	0
Smoking	29 (41.4)	11 (15.7)
**Current comorbid Axis II disorders**	22 (31.4)	0
≥ 1 current personality disorder	0 (0)	0
Cluster B borderline	14 (20.0)	0
**Family history**		
Family member with ADHD	36 (51.4)	1 (1.4)
Degree of family affected: first-degree relative(s)	26 (37.1)	1 (1.4)
Degree of family affected: first- and second-degree relative(s)	10 (14.3)	0
No answer	3 (4.3)	0
**Educational status**		
Secondary school, grade 5 to grade 9/10	37 (52.9)	7 (10.0)
University-entrance diploma, grade 12/13	12 (17.1)	40 (57.1)
Vocational qualification or other	16 (17.1)	2 (2.9)
University degree	5 (7.1)	19 (27.1)
Missing	0	2 (2.9)
**Current location**		
Village (< 5,000 inhabitants)	5 (7.1)	3 (4.3)
Town (5,000–100,000 inhabitants)	45 (64.3)	13 (18.6)
City (> 100,000 inhabitants)	20 (28.6)	51 (72.9)
Missing information	0	3 (4.3)
**Marital status**		
Unmarried	45 (64.3)	50 (71.4)
Married	20 (28.6)	16 (22.9)
Divorced	5 (7.1)	2 (2.9)
Widowed	0	0
Missing information	0	2 (2.9)
**Professional status**		
Employee	32 (45.7)	29 (41.4)
Student	15 (21.4)	34 (48.6)
Job seeker	17 (24.3)	2 (2.9)
Pensioner	4 (5.7)	1 (1.4)
Self-employed	2 (2.9)	2 (2.9)
Missing information	0	2 (2.9)

ADHD, attention-deficit/hyperactivity disorder.

**Table 2:** Logistic regression model of risk factors for anti-*T. gondii* IgG seropositivity.

	n	n (pos)	Frequency	Stepwise AIC model	
			pos in % (95% CI)	OR (95% CI)	*p* value
**Sex**					
Female	70	11	15.7 (8.1–26.4)	ref	ref
Male	70	17	24.3 (14.8–36.0)	1.69 (0.68–4.21)	0.257
**Age**	140	28	20 (13.7–27.6)	1.08 (1.02–1.14)	**0.010**
**Population of municipality**					
Village	8	2	25 (3.2–65.1)	–	–
Town	58	17	29.3 (18.1–42.7)	–	–
City	71	9	12.7 (6–22.7)	–	–
**Current cat contact**					
No	76	17	22.4 (13.6–33.4)	–	–
Yes	63	11	17.5 (9.1–29.1)	–	–
**Undercooked/raw meat consumption**					
No	68	9	13.2 (6.2–23.6)	ref	ref
Yes	71	19	26.8 (16.9–38.6)	3.06 (1.17–8.05)	**0.023**
**Body mass index (BMI)**					
Underweight (BMI < 18.5)	6	0	0 (0–45.9)	–	–
Normal weight (18.5 ≤ BMI < 25)	65	13	20 (11.1–31.8)	–	–
Overweight and obesity (≥ 25)	54	13	24.1 (13.5–37.6)	–	–
**Gardening without gloves**					
No	83	16	19.3 (11.4–29.4)	–	–
Yes	56	12	21.4 (11.6–34.4)	–	–
**Education status**					
Secondary school, grade 5 to 9/10	44	11	25 (13.2–40.3)	–	–
University-entrance diploma, grade 12/13	52	9	17.3 (8.2–30.3)	–	–
Vocational qualification or other	18	3	16.7 (3.6–41.4)	–	–
University degree	24	5	20.8 (7.1–42.2)	–	–
**Marital status**					
Never been married	95	17	17.9 (10.8–27.1)	2.49 (0.71–8.71)	0.154
Currently or previously married	43	11	25.6 (13.5–41.2)	ref	ref
**Professional status**					
Job seeking	19	6	31.6 (12.6–56.6)	–	–
Student	49	5	10.2 (3.4–22.2)	–	–
Employee or pensioner	66	16	24.2 (14.5–36.4)	–	–
Self-employed	4	1	25 (0.6–80.6)	–	–
**Total/Intercept**	140	28	20 (13.7–27.6)	0 (0–0.09)	0

AIC, Akaike information criterion; pos, seropositivity; OR, odds ratio; ref, reference category; significant results in bold.

With regard to known risk factors for seropositivity, ADHD patients more often revealed current contact with cats (n = 43 (61.4%) versus controls n = 20 (28.6%); φ = 0.33, *p* < 0.001), tended to have more direct soil contact (n = 33 (47.1%) vs. n = 23 (33.3%); φ = 0.15, *p* = 0.085), lived significantly more often in towns (φ = 0.46, *p* < 0.001) and less often in cities (φ = − 0.44, *p* < 0.001), and had significantly lower educational status than controls (secondary school, grade 5–9/10, φ = 0.46, *p* < 0.001; vocational qualification or other, φ = 0.30, *p* < 0.001). In concordance, ADHD patients were significantly less often students (φ = − 0.28; *p* < 0.001) and more frequently job seekers (φ = 0.31; *p* < 0.001). In turn, the highest education status in controls was significantly more often university-entrance diploma, grade 12/13 (φ = 0.41, *p* < 0.001), or university degree (φ = 0.27, *p* = 0.002), see Table 1. With regard to marital status, the proportions of participants who had never been married were balanced between ADHD and controls (φ = − 0.08, *p* = 0.366). The number of participants who were currently married or had ever been married was n = 25 (18.1%) in the ADHD group versus n = 18 (13%) in controls (φ = 0.11, *p* = 0.209). Rates of raw/undercooked meat consumption habits were well balanced between groups (ADHD n = 36 (51.4%) vs. controls n = 35 (50.7%); φ = 0.01, *p* = 0.866). The distribution of body mass index (BMI) revealed that ADHD patients were significantly more often overweight or obese with a BMI above 25 (φ = 0.23; *p* = 0.006). In contrast, controls had significantly more often spent several months abroad (n = 29 (42.6%) vs. ADHD n = 10 (14.9%); φ = 0.25, *p* = 0.002).

#### Seropositivity and ADHD

Out of 140 individuals, 20% (n = 28) were seropositive for anti-*T. gondii* IgG, 0% for IgM. In the ADHD group, 27.1% (n = 19) were seropositive for anti-*T. gondii* IgG versus 12.9% (n = 9) in the control group. ADHD showed a significant association with anti-*T. gondii* seropositivity in the unavailable analysis (odds ratio (OR) 2.53; 95% confidence interval (CI) 1.05–6.06; *p* = 0.038). This was confirmed by confounder-adjusted multivariable analysis (OR 2.77; 95% CI 1.013–7.56; *p* = 0.047).

#### Risk factors for seropositivity

In order to examine an association between potential risk factors for *T. gondii* seropositivity, a multiple logistic regression was performed (see Table 2). By applying a stepwise analysis, male sex, age, never been married, and raw/undercooked meat consumption were found to be associated with *T. gondii* seropositivity with regard to the entire study sample. Other less-fitting covariates were eliminated following the Akaike information criterion (AIC) model selection. The variable living abroad was excluded from analysis, as only four seropositive participants lived abroad for a longer period. Among them, only one participant had ADHD. These case counts were too low to allow further analysis.

Similarly, a separate regression analysis of risk factors for seropositivity in the ADHD group yielded a model in which male sex (OR 3.00; 95% CI 0.86–10.45, *p* = 0.085), age (OR 1.11; 95%-CI 1.02–1.2, *p* = 0.015), never been married (OR 3.75; 95%-CI 0.71–19.69, *p* = 0.118), and raw/undercooked meat consumption (OR 4.26; 95%-CI 11.13–16.06, *p* = 0.032) were predictive for seropositivity of *T. gondii*.

#### *Toxopasma gondii* seropositivity

**Figure 1:**
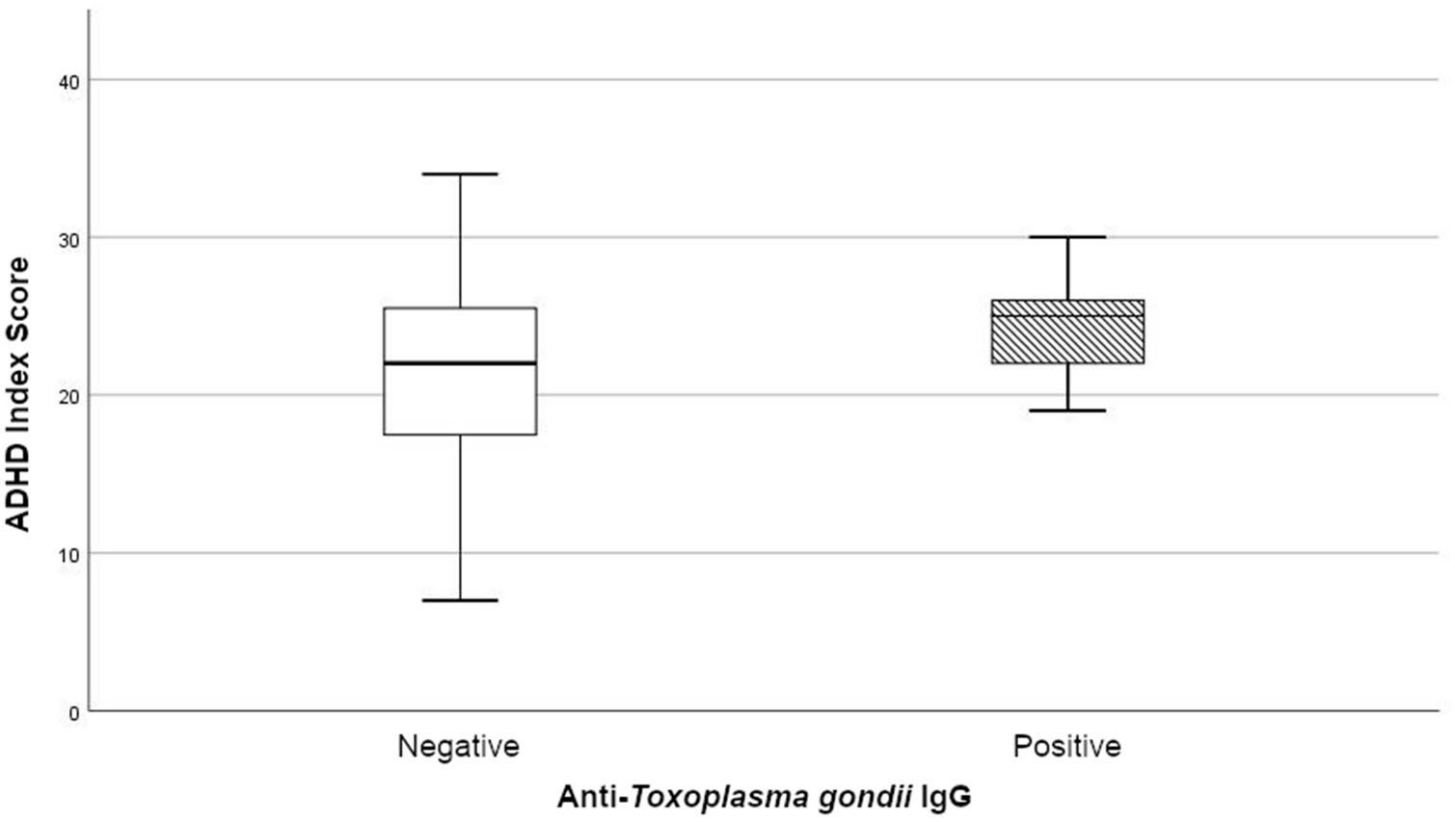
Comparison of ADHD symptom severity: anti-*T. gondii* IgG-positive (m = 24.58, SD: 3.29) and IgG-negative (m = 21.12, SD: 6.1) subjects. Box plot of the Conners' Adult ADHD Rating Scale ADHD Index in the ADHD group (n = 70). The lower and upper box boundaries show the 25th and 75th percentiles, respectively; the line inside box depicts the median; the box contains the middle 50% of recorded data; the error bars display the minimum and maximum values. A significant influence of *T. gondii* was found in linear regression models. IgG, immunoglobulin G.

In order to examine the impact of *T. gondii* seropositivity on ADHD symptom severity, multiple linear regression models were constructed, which revealed significant influences of *T. gondii* seropositivity on ADHD-related symptoms. Seropositivity showed a significant association with ADHD-related symptoms, represented by the ADHD Index, with regard to the entire sample (Table 3) and the ADHD group (unstandardized coefficient (B) = 3.60; 95%-CI 0.83–6.37, *p* = 0.012) (Supplementary Table 3). Patients with adult ADHD and *T. gondii* antibodies showed significantly higher ADHD Index scores than seronegative ADHD patients (see Fig. 1).

**Table 3:** Linear regression model of *T. gondii* seropositivity and ADHD Index, all cases.

Minimal model (n = 139)	ADHD Index (adjusted R^2^ = 0.050)			
		95% CI		
	B	Lower	Upper	*p* value
(Intercept)	13.29	11.60	14.97	**< 0.001**
*T. gondii* (pos)	5.46	1.71	9.22	**0.005**

Final model (n = 139)	ADHD Index (adjusted R^2^ = 0.742)			
		95% CI		
	B	Lower	Upper	*p* value
(Intercept)	6.22	5.07	7.37	**< 0.001**
*T. gondii* (pos)	2.14	0.14	4.13	**0.037**
ADHD	14.13	12.38	15.87	**< 0.001**
BPD	4.43	1.55	7.30	**0.003**
Anxiety disorder	2.34	− 0.24	4.93	0.076
Hypnotics, sedatives	− 4.30	− 10.00	1.41	0.139

ADHD Index, self-rated Conners' Adult ADHD Rating Scale ADHD Index, long version; *T. gondii*, *Toxoplasma gondii*; pos, seropositive; ADHD, attention-deficit/hyperactivity disorder; BPD, borderline personality disorder; significant results in bold.

#### *Toxopasma gondii* serointensity

When linking *T. gondii* with psychiatric diseases, prior research demonstrated the importance of serointensity over the measurement of seroprevalence^46^. Therefore, further analyses were performed taking *T. gondii* serointensity into account. The mean IgG concentration level of all *T. gondii* seropositive study participants was m = 79.0 U/ml (SD: 60.0 U/ml, minimum 14 U/ml, maximum 248 U/ml).

**Figure 2:**
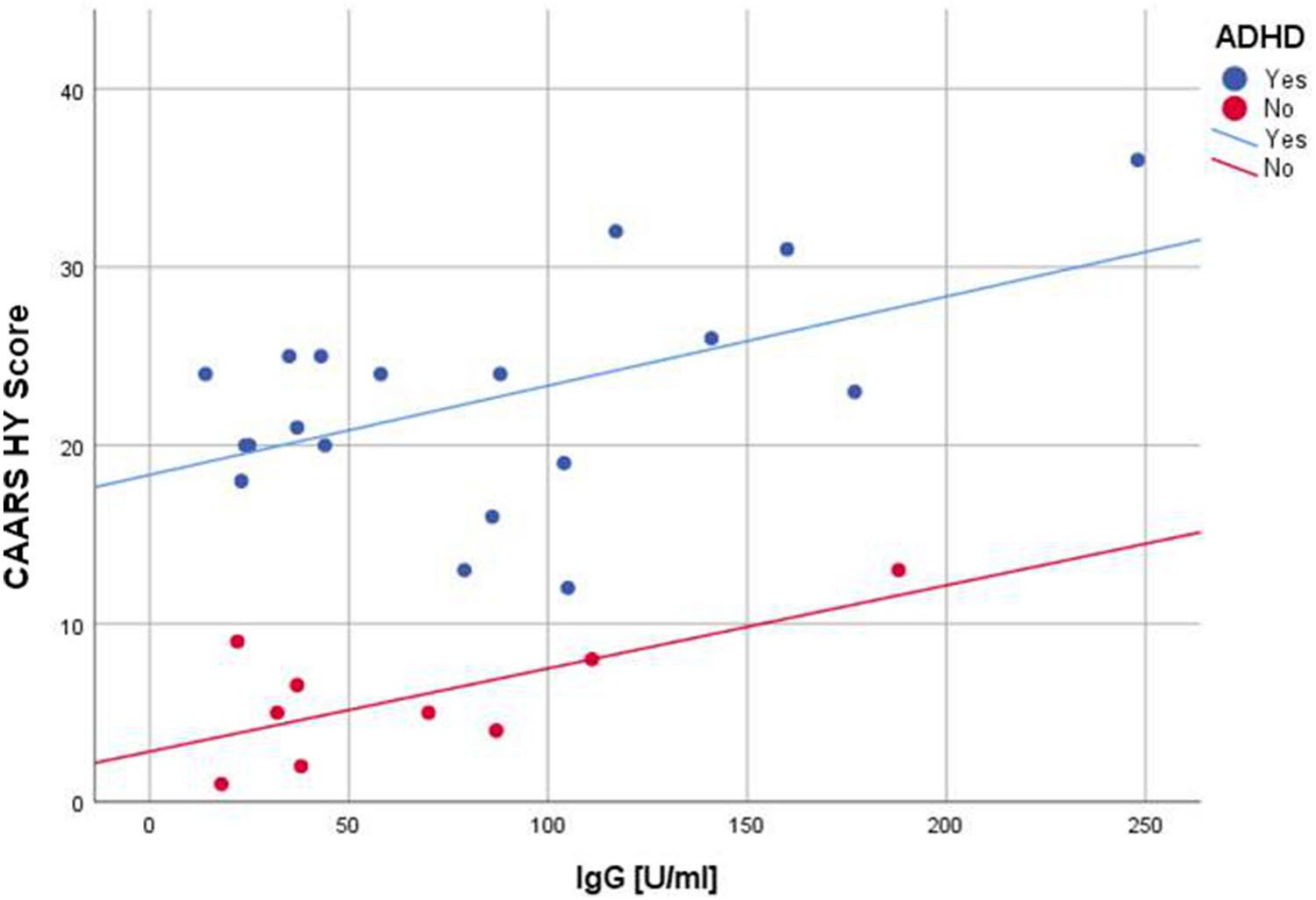
Regression analysis of anti-*T. gondii* IgG and hyperactivity in ADHD patients versus controls. Yes: R^2^ = 0.263; No: R^2^ = 0.485. CAARS HY Score, hyperactivity/restlessness subscale of Conners' Adult ADHD Rating Scale; IgG, anti-*T. gondii* immunoglobulin G; ADHD, attention-deficit/hyperactivity disorder.

Ordinary linear regression models revealed a positive association between serointensity and the hyperactivity subscales in subjects with and without ADHD (Fig. 2).

**Figure 3:**
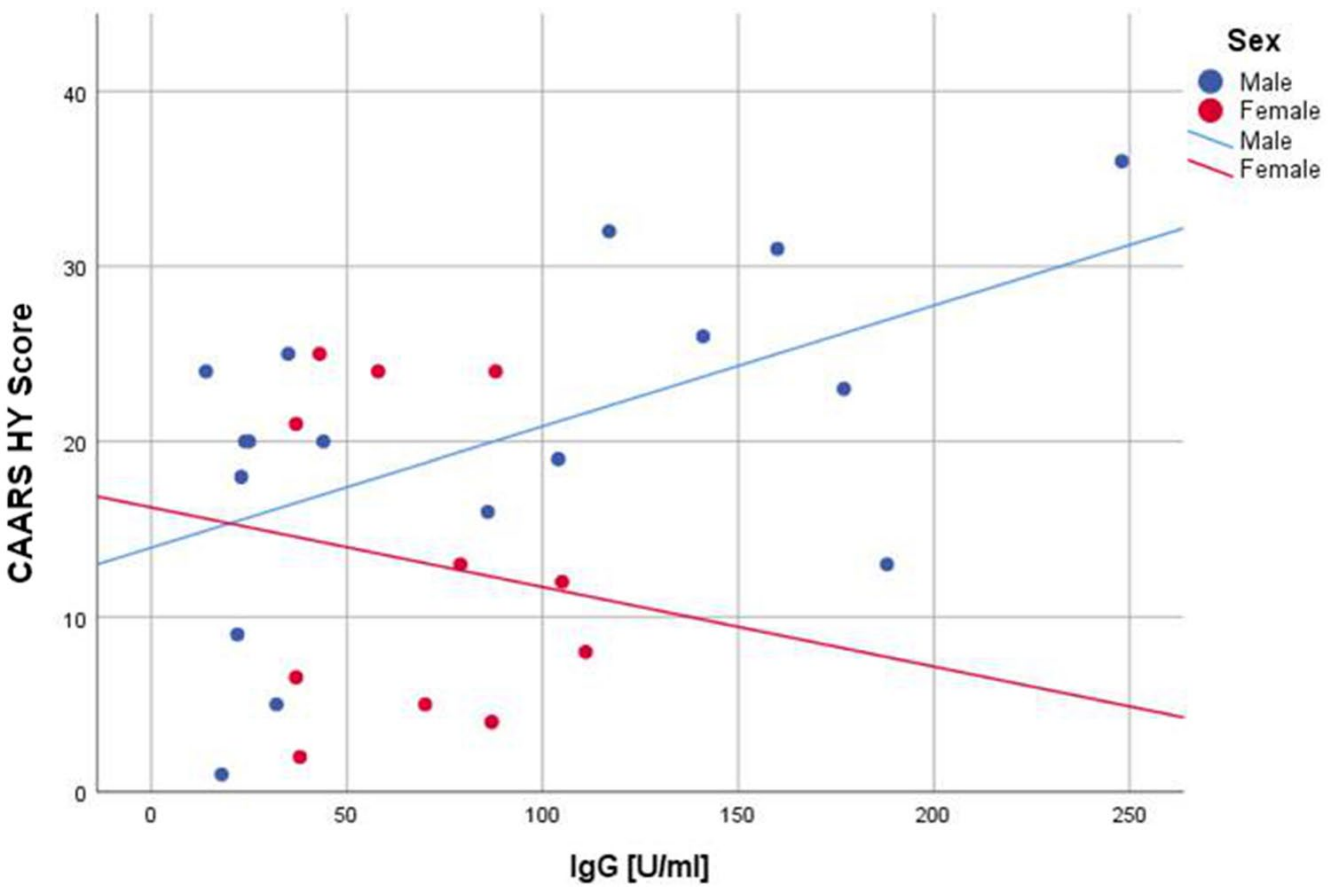
Regression analysis of anti-*T. gondii* IgG and hyperactivity in males versus females. Male: R^2^ = 0.301; Female: R^2^ = 0.020. CAARS HY Score, hyperactivity/restlessness subscale of Conners' Adult ADHD Rating Scale; IgG, anti-*T. gondii* immunoglobulin G; ADHD, attention-deficit/hyperactivity disorder.

In sex-differentiated bivariate regression analysis, the highest coefficients of determination (R^2^) were found for hyperactivity. As a result, seropositivity demonstrated a positive association with hyperactivity in males as measured by the Conners' Adult ADHD Rating Scale (CAARS) hyperactivity subscale, while females revealed a weak negative association with a low R^2^ value (Fig. 3).

To clarify to what extent ADHD symptoms were influenced by serointensity, multiple linear regression models were estimated (Tables 4 and 5 and Supplementary Tables 4 and 5). In minimal models, serointensity showed a significant association with the ADHD Index and all subscales except for problems with self-concept (CAARS SKP), Supplementary Table 4. In the final models, serointensity had significant influences on the ADHD Index and the ADHD Total score according to the Diagnostic and Statistical Manual of Mental Disorders Fourth Edition (DSM-IV) as well as on both hyperactivity subscales (CAARS HY and DSM-HY/I), Tables 4 and 5. Sex showed significant influences on hyperactivity (CAARS HY) and inattention/memory problems (CAARS UA), Table 4 and Supplementary Table 5. Current pharmacological ADHD treatment showed a significant negative association with hyperactivity (CAARS HY), Table 4. The covariate ‘anxiety disorder’ remained in all final models of the self-rated long version of CAARS (CAARS-S:L) after backward variable elimination. ‘Affective disorders’ had also been entered in all variable selection processes during data analysis but were not retained in the final models of CAARS-S:L and its subscales after AIC-based model selection. Moreover, stepwise regression analysis of the metric responses of the BDI-II questionnaire did not show a statistical association between depression and either *T. gondii* seropositivity or *T. gondii* serointensity (data not shown).

**Table 4:** Stepwise regression analysis of serointensity and CAARS scores, all cases.

Minimal model (n = 139)	ADHD Index (adjusted R^2^ = 0.041)				CAARS HY (adjusted R^2^ = 0.079)			
		95% CI				95% CI		
	B	Lower	Upper	*p* value	B	Lower	Upper	*p* value
(Intercept)	13.61	11.99	15.23	**< 0.001**	12.78	11.32	14.24	**< 0.001**
IgG [U/ml]	0.05	0.01	0.09	**0.010**	0.06	0.03	0.09	**0.001**

Final model (n = 139)	ADHD Index (adjusted R^2^ = 0.743)				CAARS HY (adjusted R^2^ = 0.601)			
		95% CI				95% CI		
	B	Lower	Upper	*p* value	B	Lower	Upper	*p* value
(Intercept)	6.31	5.18	7.44	**< 0.001**	6.21	4.64	7.78	**< 0.001**
IgG [U/ml]	0.02	0.00	0.04	**0.035**	0.04	0.02	0.06	**0.001**
ADHD	14.06	12.30	15.82	**< 0.001**	11.81	9.29	14.32	**< 0.001**
BPD	4.68	1.79	7.57	**0.002**	3.11	− 0.21	6.43	0.066
Gender: male	–	–	–	–	2.07	0.19	3.95	**0.031**
Anxiety disorder	2.50	− 0.09	5.09	0.058	3.94	0.95	6.93	**0.010**
ADHD medication	–	–	–	–	− 3.74	− 6.35	− 1.13	**0.005**
Antidepressants	–	–	–	–	–	–	–	–
Hypnotics. sedatives	− 4.49	− 10.18	1.20	0.121	–	–	–	–
Eating disorder	–	–	–	–	5.72	− 0.64	12.08	0.077

ADHD Index, self-rated Conners' Adult ADHD Rating Scale ADHD Index, long version; HY, hyperactivity/restlessness; IgG, immunoglobulin G; ADHD, attention-deficit/hyperactivity disorder; BPD, borderline personality disorder; significant results in bold.

**Table 5:** Stepwise regression analysis of serointensity and CAARS scores, all cases.

Minimal model (n = 139)	DSM-ADHD Total (adjusted R^2^ = 0.060)				DSM-HY/I (adjusted R^2^ = 0.078)			
		95% CI				95% CI		
	B	Lower	Upper	*p* value	B	Lower	Upper	*p* value
(Intercept)	17.37	15.03	19.72	**< 0.001**	7.46	6.29	8.63	**< 0.001**
IgG [U/ml]	0.08	0.03	0.14	**0.002**	0.05	0.02	0.07	**0.001**

Final model (n = 139)	DSM-ADHD Total (adjusted R^2^ = 0.761)				DSM-HY/I (adjusted R^2^ = 0.654)			
		95% CI				95% CI		
	B	Lower	Upper	*p* value	B	Lower	Upper	*p* value
(Intercept)	3.15	− 0.62	6.92	0.101	0.55	-1.74	2.84	0.635
IgG [U/ml]	0.04	0.02	0.07	**0.002**	0.03	0.01	0.05	**< 0.001**
ADHD	18.93	16.30	21.57	**< 0.001**	7.69	6.10	9.29	**< 0.001**
BPD	5.91	1.77	10.06	**0.006**	4.63	2.11	7.14	**< 0.001**
Age	0.12	0.01	0.23	**0.040**	0.07	0.00	0.14	**0.042**
Anxiety disorder	5.48	1.76	9.21	**0.004**	2.86	0.61	5.12	**0.013**
Substance abuse (lifetime)	2.62	− 1.07	6.32	0.162	1.78	− 0.46	4.02	0.118

CAARS, self-rated Conners' Adult ADHD Rating Scale, long version; DSM-ADHD Total, ADHD symptoms according to DSM; DSM-HY/I, hyperactivity/impulsivity according to DSM; IgG, immunoglobulin G; ADHD, attention-deficit/hyperactivity disorder; BPD, borderline personality disorder; significant results in bold.

#### *Toxopasma gondii* avidity

**Figure 4:**
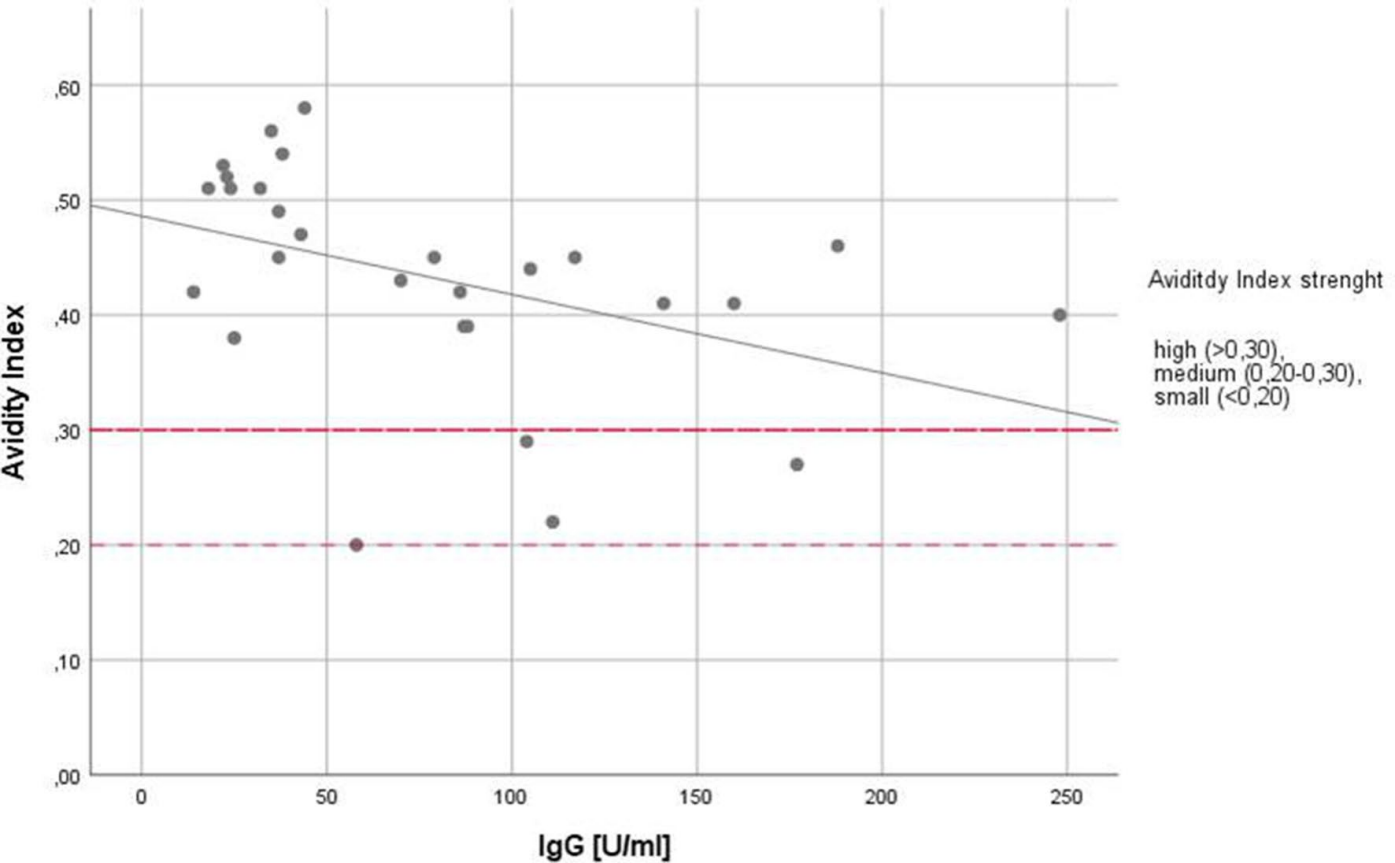
Avidity index of anti-*T. gondii* IgG. R^2^ = 0.183. IgG, anti-*T. gondii* immunoglobulin G**.**

The mean avidity index was m = 0.43 (SD: 1.0, minimum 0.2, maximum 0.6), with one individual having a small avidity index strength of < 0.20, three individuals having a medium avidity index strength and the majority (n = 24) showing a high avidity index strength of > 0.30. Avidity and serointensity showed a significant negative correlation (n = 28; r_s_ = − 0.543; *p* 0.003; Fig. 4). Multiple linear regression analysis revealed a significant influence of *T. gondii* avidity on ADHD-related symptoms (Table 6). Avidity was significantly associated with increased symptom severity in the ADHD group, as demonstrated by elevated ADHD Index scores (B = 7.98; 95%-CI 1.65–14.32, *p* = 0.014), Supplementary Table 6.

**Table 6:** Linear regression model of avidity and ADHD Index, all cases.

Minimal model (n = 139)	ADHD Index (adjusted R^2^ = 0.042)			
		95% CI		
	B	Lower	Upper	*p* value
(Intercept)	13.39	11.71	15.08	**< 0.001**
Avidity	11.41	2.93	19.89	**0.009**

Final model (n = 139)	ADHD Index (adjusted R^2^ = 0.741)			
		95% CI		
	B	Lower	Upper	*p* value
(Intercept)	6.24	5.09	7.39	**< 0.001**
Avidity	4.40	− 0.09	8.89	0.055
ADHD	14.20	12.46	15.94	**< 0.001**
BPD	4.28	1.40	7.17	**0.004**
Anxiety disorder	2.40	− 0.20	4.99	0.070
Hypnotics, sedatives	− 4.29	− 10.02	1.43	0.140

ADHD Index, Conners' Adult ADHD Rating Scale ADHD Index, long version; ADHD, attention-deficit/hyperactivity disorder; BPD, borderline personality disorder; significant results in bold.

## Discussion

This case–control study is the first to show that seropositive individuals had more than 2.5 times the adult ADHD risk of seronegative individuals. Two previous studies from Iran conducted with children and adolescents found no significant differences in *T. gondii* seropositivity between subjects with and without ADHD^47,48^. Nevertheless, more children and adolescents with clinically severe ADHD than mild or moderate ADHD were shown to have antibodies against *T. gondii*^47^. These findings had not been further evaluated with regard to the different aspects of ADHD symptoms. Another study including 188 patients at a mean age of 16.8 years (SD: 7.0), carried out in Egypt, reported a significant association of *T. gondii* seropositivity with neurodevelopmental disorders among fourteen subjects with ADHD^13^. This cited study is limited to the small sample size of ADHD patients, the lack of a control group, and the lack of inclusion of risk factors for seropositivity. Finally, the preceding studies did not investigate the impact of *T. gondii* serointensity^49^ or IgG avidity on the clinical course of ADHD.

The seroprevalence of *T. gondii* in humans varies between 1 and 100% throughout different countries, depending on eating habits, hygiene standards, health behavior, age, culture, geographic and climatic factors^14,15,23–25,50–53^. For Germany, an age-related prevalence of IgG antibodies has recently been found, indicating a seroprevalence of 20% (95%-CI 17–23%) in the group of 18–29 years of age and 77% (95%-CI 73–81%) in the group of 70–79 years of age^22^. In concordance with the previous findings, our study, which was also conducted in Germany, revealed a seropositivity of 20% in a study population of 31.8 years on average (SD: 10.4).

Associations between epidemiological aspects and the higher seropositivity of *T. gondii* in the ADHD group were investigated by integrating identified risk factors for seropositivity into our analyses. The main risk factors in humans are consuming the parasite’s cysts through cyst-carrying undercooked meat, oocyst-contaminated soil, or contact with fecal material of infected cats^22^. Moreover, a previous population-based investigation on *T. gondii* seroprevalence demonstrated that an older age, male sex, BMI > 25, low socioeconomic status, living in rural or moderately urbanized areas, and marital status were associated with *T. gondii* seropositivity^22,54^. In Germany, eating habits, particularly consuming raw meat, were found to be of high epidemiological relevance^22^. The higher seroprevalence observed in men has been discussed as a result of eating habits, as male Germans are known to eat approximately twice as much meat as females, which in turn contributes to an elevated risk of ingesting contaminated meat^22^. Our results are in line with these findings, as the presented data confirm male sex, age, marital status, and consumption of raw/undercooked meat as predictors for *T. gondii* seropositivity. Cat contact, BMI, municipality size, and low educational or professional status were not associated with seropositivity in our analysis. The risk for infection of cat owners has been controversial^22^. A previous study suggested an elevated risk for individuals living with three or more cats^55^. This specific question was not addressed in the questionnaire used in our study. Last, we did not evaluate lifetime cat contact or whether the individuals had contact with cat litter, which has been shown to be associated with an elevated risk of infection^22^.

To our knowledge, our results suggest an impact of *T. gondii* seropositivity on the clinical course of adult ADHD and reveal specific influences of *T. gondii* serointensity and IgG avidity on the severity of ADHD symptoms for the first time. The influences were found in subjects with ADHD and controls, which hints at the fact that latent *T. gondii* infection might be able to trigger ADHD-related symptoms in individuals without ADHD. Above all, the present study illustrates an aggravation of symptoms in ADHD patients, especially in males, which was dependent on the serum levels of *T. gondii* antibodies and the strength of the antibody-binding avidity.

Previous animal models and behavioral studies in humans have indicated that latent toxoplasmosis may lead to behavioral changes^24–27^. Indeed, our finding of increased hyperactivity in *T. gondii*-seropositive participants correspond with behavioral changes found in *T. gondii*-infected rodents, which also showed higher activity levels than uninfected controls^38,56–58^. The underlying mechanism by which *T. gondii* manipulates brain function is not yet fully understood. Experimental studies in mice revealed neurological and behavioral abnormalities following inflammation and loss of brain parenchyma due to *T. gondii* infection^59^. Moreover, several behavioral changes in rodents have been linked to changes in the dopaminergic pathway of various origins: local immune responses due to infection with the parasite (e.g., proinflammatory cytokines, indoleamine 2,3-dioxigenase, interferon-c) cause modifications of the turnover, efficiency, and levels of neuromodulators such as dopamine, serotonin, and glutamate^16,27,60–62^. Finally, *T. gondii* showed its capability to directly influence neurotransmitter levels by enhancing the release of dopamine from neurons in vitro and producing dopamine in vivo with its own genes encoding tyrosine hydroxylase, which represents a rate-limiting enzyme of dopamine biosynthesis^60^. In infected mice, *T. gondii* cysts were found throughout the brain with higher percentages in the amygdala, nucleus accumbens^32^, and hypothalamus^63^. The amygdala and the nucleus accumbens, as dopamine-containing brain regions, are well known to have important functions in the control of movements (basal ganglia) and fear (amygdala), respectively^16,32^. Altered dopamine levels induced by *T. gondii* are discussed to have functional consequences leading to behavioral changes and neurological dysfunction^26^. Previous studies found that the manipulation patterns vary among the three existing *T. gondii* strains due to different prevalence, virulence, and neuropathogenic potential^15,47,64^. Only infections with the type I strain showed abnormalities in the three neurotransmitter systems (dopamine, glutamate, and serotonin) and two neuropeptides (PROK2 and TAC1) compared to controls^31^. This may be one explanation for the heterogeneity of *T. gondii*-associated neurological changes^31^ and should therefore be taken into account in future studies on ADHD and *T. gondii.*

Additionally, *T. gondii* has been found to interact with approximately 3000 host genes or proteins during its life cycle, including 18% of ADHD susceptibility genes in humans with a specific emphasis on the calcium signaling and neurotransmitter pathways (dopaminergic, cocaine addiction, and ligand/receptor interactions)^30^. Moreover, overlapping susceptibility genes of ADHD and *T. gondii* in various metabolic pathways, e.g., phenylalanine, tyrosine, tyrosine, histidine, and unsaturated fatty acid synthesis, were found^30^. Furthermore, *T. gondii* was linked to increased expression of miR-132 in mice, which led to reduced expression of D1-like dopamine receptors (DRD1, DRD5), metabolizing enzyme monoamine oxidase A (MAOA), and several intracellular proteins linked to the transduction of dopamine-mediated signaling^31^. In ADHD, changed levels of peripheral miRNA can be found in both ADHD animal models and humans^34^. Furthermore, miRNAs, as posttranscriptional regulators, have been shown to modulate the gene expression of several genes linked to ADHD etiology, e.g., dopamine transporter (DAT1)^65–67^. As a consequence, changes in the dopaminergic pathway, which have been shown by both *T. gondii* and attention-deficit/hyperactivity disorder, might display the link to the clinical alterations in *T. gondii*-seropositive adults with ADHD seen in our study.

In prior research, a stronger effect of *T. gondii* on human behavior was found with older age and longer duration of infection, assessed via IgG avidity^24,50,68^. While in our study seropositivity was associated with stronger ADHD-related symptoms in the whole sample, avidity had a significant influence on the ADHD Index only in ADHD patients. This result suggests a stronger impact of a latent infection on the severity of ADHD symptomatology in ADHD-affected individuals which increases with the duration of the infection. Thus, it can be assumed that a longer infection period enables the parasite to be more likely to influence the host’s neuromodulators, immunity, or hormonal system^60,61,69–74^. Two previous studies discussed the impact of the timing of infection with *T. gondii* on seropositivity among children with ADHD^47,48^. Although both studies were conducted in the same country, the seropositivity found in children and adolescents was not in line with available data on the prevalence of *T. gondii* in their country, suggesting greater parasite exposure in later life^48^. It has been remarked that the maturation of the IgG response varies noticeably between individuals^75^. Although it remained unclear whether a linearity of the avidity-maturation curve over time can be assumed, the antibody-binding avidity increases over time and therefore provides additional information about the age of an infection^44,75^. High avidity index strength was measured in 86% of participants in our study (see Fig. 4). Additionally, anti-*T. gondii* IgM was negative in all participants. Altogether, the presence of new infections with *T. gondii* could be excluded in the study sample. As we provide data of an association of *T. gondii* IgG avidity, which reflects the duration of infection, with aggravation of symptoms in adult ADHD, the factor time should be considered in future studies. Our findings could pave the way for further investigations on the influence of the parasite over time on neurodevelopmental alterations linked to ADHD pathophysiology.

Previous studies in humans with latent *T. gondii* infection found differential effects on several behavioral traits according to sex^24,25,50^. In line with these discoveries, we found a positive association of hyperactivity and serointensity in males. In infected females, this association tends to be oppositional and needs to be further evaluated in a larger sample size. However, this is the first study showing a sex-dependent effect of *T. gondii* serointensity on ADHD symptomatology. It has been mentioned in the literature that the observed behavioral changes in *T. gondii*-infected humans and rodents in general could be linked to changed testosterone levels induced by the parasite, as high steroid hormone levels have been associated with lower immunity and make the persistence of the parasite more likely^24^. Former studies in humans reported an increased concentration of testosterone in men with latent toxoplasmosis compared to *Toxoplasma*-negative individuals, while an opposite direction of the testosterone shift was found in women^69–72^. Future studies are needed to investigate the clinical impact of testosterone in *T. gondii*-infected subjects with ADHD.

Our study was conducted at a time when the current meta-analysis found no significant association between *T. gondii* and depression^18,76^. Therefore, we did not exclude ADHD patients with comorbid depressive disorders during patient recruitment. As the association of *T. gondii* in neuropsychiatric disorders has received increasing research interest, several new studies have been conducted. According to recent meta-analysis findings, *T. gondii* does not display a risk factor for major depressive disorder (MDD), while associations with dysthymia as well as mild and moderate depression have been found^77^. However, our data did not reveal an influence of *T. gondii* seropositivity or serointensity on depressive symptoms.

Current discussions point out that environmental risk factors may alter the subsequent course of ADHD^78^. Our study suggests that *T. gondii* should be regarded as one of these environmental risk factors for ADHD in adulthood that may contribute to symptom severity. Our data hints at the fact that the pathomechanism of a latent *T. gondii* infection, in addition to an affected dopaminergic system in ADHD patients, may lead to an aggravation of ADHD symptoms, especially contributing to increased hyperactivity. Despite the high *T. gondii* seropositivity found, the presented results should be judged with care, as our data are based on a case–control study and are not population-based. Discussions on causative or influencing effects should consider additional factors, which are generally difficult to assess, e.g., individual immunity and resistance of the host, timing and severity of infection, epigenetics and confounding medical and environmental factors^30^. Additional studies are required to clarify the underlying neurobiological mechanisms of our clinical findings. Moreover, the impact of *T. gondii* on the course of ADHD symptomatology in the long term should be taken into account in future research with regard to prevention, diagnostic proceedings, and therapeutic implications. Further longitudinal studies are needed to investigate possible differences in the pharmacological response or treatment outcome of *T. gondii* seropositive versus seronegative ADHD patients. Different psychopharmacological medications like the antipsychotic dopamine receptor agonist haloperidol, the mood stabilizer valproic acid, or the selective dopamine reuptake inhibitor vanoxerine were found to inhibit replication in vitro or inhibit the behavioral effects of *T. gondii* in rodents^61,79,80^. With regard to these experimental findings, potential interactions of methylphenidate and *T. gondii* require exploration. The norepinephrine-dopamine reuptake inhibitor methylphenidate is the recommended first-line treatment for ADHD^81^. However, the response to this medication as well as long-term efficacy can vary among individuals^82,83^. Studies are warranted to clarify whether the effect of methylphenidate might be reduced or even augmented through a co-occurring latent *T. gondii* infection. On the one hand, methylphenidate should in turn be investigated for its ability to influence parasite replication, and on the other hand, it is worthwhile to explore whether *T. gondii* seropositive patients with ADHD profit from dopamine-enhancing medication.

Our results provide support for the hypothesis of an association between latent *T. gondii* infection and the symptom severity of ADHD patients. Our findings suggest that *T. gondii* might display a disregarded environmental influencing factor of ADHD in adulthood.

## Methods

### Study design and setting

In this clinic-based case–control study, patients were consecutively recruited from the University Hospital of Psychiatry and Psychotherapy at the Carl von Ossietzky University of Oldenburg, European Medical School Oldenburg in Germany. The hospital was running specialized psychiatric and psychotherapeutic ADHD inpatient and outpatient units. Medical staff as well as research facilities were broadly experienced in the assessment and treatment of ADHD in adulthood. The controls were recruited via announcements at the website of the same university. In addition, the mention of toxoplasmosis on the website was omitted to avoid overrepresentation of groups of people who may have a particular interest in an antibody test against *T. gondii* or who are particularly familiar with the subject (e.g., pregnant women, cat owners, etc.). Recruitment was performed between May 2016 and November 2017. Laboratory analyses were performed at the Institute for Laboratory Diagnostics and Microbiology at the Klinikum Oldenburg. Anti-*T. gondii* avidity was analyzed at the Medical Laboratory in Bremen. The study received ethics committee approval from the local ethics committee (Faculty of Medicine, University of Oldenburg, 2016–009). All methods were performed in accordance with the relevant guidelines and regulations. Written informed consent was obtained from all included subjects before study participation.

### Sample size

To detect a higher *T. gondii* seropositivity with an OR of 2.5 between adult ADHD patients and healthy controls, a minimum power of 80%, and a significance level of 5% in the logistic regression, 140 participants were needed.

### Assessment for eligibility

#### Inclusion and exclusion criteria

We included sex- and age-matched participants, if at least 18 years old, who spoke and understood German and lacked clinically significant abnormalities detected on physical examination or blood samples. Patients were required to fulfill the criteria for ADHD according to DSM-IV with a chronic course of ADHD symptoms from childhood to adulthood. Only patients with the combined subtype of ADHD were included.

Exclusion criteria for patients and controls were acute severe inflammation or infection, detected via CRP; excluded ≥ 5 mg/dl) and differential blood count, unwillingness or incapability to adhere to the study protocol, comorbid neuropsychiatric disorders known to be associated with *T. gondii* (schizophrenia, bipolar disorder, autism, obsessive compulsive disorder, Parkinson’s disease), treatment with stimulants or ADHD-specific medication that cannot be terminated three days prior to blood sampling and questionnaires, pregnancy or breast-feeding, and severe abnormality known or detected on routine blood testing (i.e., thyroid dysfunction).

Moreover, controls were excluded if they had any psychiatric disorder except tobacco dependency or specific phobia, as no problematic relationship between these disorders and *T. gondii* is assumed.

All participants took part in the full diagnostic process and received 10 Euros as monetary compensation. In the diagnostic process, we used diagnostic interviews as well as self-rating scales in German. The diagnosis (or exclusion) of ADHD in adulthood and other psychiatric disorders were established by psychiatric expert assessment and validated using instruments such as the ADHD Self-Rating Scale (ADHD-SR, German Version)^84^ and the Wender-Utah-Rating-Scale for the retrospective assessment of ADHD in childhood (WURS-k^85^).

To assess further disorders, the structured clinical interviews for DSM-IV (SCID-I, SCID-II; covering Axis I and personality disorders^86^), the self-report inventory for the assessment of depression (Beck Depression Inventory (BDI-II^87^), revised version 1996), and a self-rating form to assess autistic symptoms [Autismus Spektrum Quotienten (AQ^88^)] were used.

Current ADHD symptoms were assessed via the CAARS-S:L^89–91^. The CAARS provides a balanced assessment of adult ADHD symptoms in different areas of life and indicates more severe symptoms by higher values. The severity of ADHD-related symptoms was measured via the ADHD Index of CARRS-S:L. Subscales assessed ADHD-related symptoms and behavior cross-sectionally (inattention/memory problems (CAARS UA), hyperactivity (CAARS HY), impulsivity/emotional instability (CAARS I/EL), and problems with self-concept (CAARS SKP)). Three further subscales corresponding to the DSM-IV were also included (inattention/memory problems according to DSM-IV (UA-DSM), ADHD symptoms according to DSM (ADHD Total score), and hyperactivity/impulsivity according to DSM (DSM-HY/I)).

All applied instruments were tested for reliability and validity (Supplementary Table 1).

Additionally, a specific questionnaire was administered capturing data on socioeconomic status, medical history, educational background of participants, and known behavioral risk factors for *T. gondii* infection. The education level of the participant was measured as the highest degree completed by the individual. Smoking and substance abuse were coded as ‘current’ versus ‘past’ or ‘never’. Cat contact was coded as ‘current contact’ or ‘contact in the past’. Soil contact by gardening was coded as ‘direct skin contact’, ‘no skin contact due to gloves’, or ‘no soil contact at all’.

### Laboratory testing

Venous blood samples were taken from all eligible participants. Concurrent intake of ADHD medication was discontinued at least three days prior to the diagnostic assessment.

All blood samples were tested for *T. gondii* IgG and IgM antibodies^92^ using the enzyme immunoassay kit Enzygnost Toxoplasmosis IgG and IgM (Siemens Healthcare Diagnostics Products GmbH, Marburg, Germany). Serological assays were performed on the automated BEP 2000 system (Siemens Healthcare Diagnostics Products GmbH, Marburg, Germany), and *T. gondii* antibody titers were categorized as negative (< 6 U/ml) or positive (> 6 U/ml), according to the instructions of the manufacturer. In seropositive respondents, a subanalysis of the serointensity was performed. Concentrations of anti-*T. gondii* IgG were obtained in units per milliliter (U/ml). We further tested the avidity of *T. gondii* antibodies by using the fully automated chemiluminescence analyzer LIAISON XL (DiaSorin S.p.A. Via Crescentino, snc, Saluggia (VC) Italy). The avidity index allows specimen classification as low (avidity index, < 0.2), moderate (avidity index, 0.20 to 0.30), or high (avidity index, > 0.30). In addition, the CRP concentrations were determined using an immunoturbidimetric assay on a Cobas 6000 analyzer system (Roche Diagnostics GmbH, Germany). Differential blood counts were performed using an Advia 2120-System (Siemens Healthcare Diagnostics, Deerfield, IL). Blood serum aliquots of each sample were stored at − 80 °C for testing of *Toxoplasma* IgG avidity, which was conducted after completion of all blood sample collections.

### Statistical analysis

A descriptive analysis was performed for all variables included in the analyses. Frequencies are reported for categorical variables. For continuous variables, the mean and standard deviation were calculated. Risk factors for anti-*T. gondii* IgG seropositivity between groups were compared by Pearson’s chi-squared test.

In the main analysis, a logistic regression model was used with *T. gondii* seropositivity as the dependent variable to assess the crude and confounder-adjusted OR for the association between *T. gondii* seropositivity and ADHD. Covariates were taken into account as confounders based on a priori hypotheses considering covariates associated with anti-*T. gondii* seropositivity. The fully adjusted model included sex, age, education status, marital status, professional status, current cat contact, soil contact by gardening without gloves, and raw/undercooked meat consumption. The results are presented as ORs with 95% CIs.

In order to investigate associations between explanatory variables of infection and anti-*T. gondii* IgG seropositivity, a logistic regression model was developed. Multivariable regression analyses were performed by using backward variable elimination based on the AIC. Risk factors were included as covariates with *T. gondii* seropositivity as the dependent variable. The following covariates including the reference category (ref) were set: age (in years), sex (ref ‘female’), BMI (ref ‘normal weight’), education status (ref ‘university-entrance diploma’), municipality size (ref ‘city’), marital status (ref ‘currently or previously married’), professional status, (ref ‘job seeker), current cat contact (ref ‘no’), soil contact by gardening without gloves (ref ‘no’), raw/undercooked meat consumption (ref ‘no’), and living abroad (ref ‘no’). A second model was developed for the analysis of ADHD patients only.

Age was self-reported. Marital status was dichotomized as ‘never married’ and ‘currently or previously married’ due to the small sample size of divorced and widowed participants. Professional status was categorized as ‘job seeker’, ‘student’, ‘employee or pensioner’, and ‘self-employed’. The BMI was included as studies indicate that a BMI > 25 might be a risk factor for seropositivity^22^. At the same time, ADHD patients are known to suffer from a higher BMI than controls^93^. As the seroprevalence varies throughout different countries between 1 and 100%, depending on eating habits, hygiene standards, and health behavior^51–53^, we assessed whether the participants had already spent (or lived) several months abroad during their lifespan, defined as more than 4 weeks. The results are presented as ORs with 95% CIs.

In secondary analyses, the association between IgG and ADHD symptom severity was estimated. This association was explored in scatter plots with bivariate linear regression models of the ADHD group and sex. Moreover, linear regression models were constructed for the metric responses of the CAARS-S:L^89–91^ questionnaires. For each linear regression model, a separate stepwise backward variable elimination based on the AIC was performed starting with all predictors in a full model. Based on the AIC, one term was removed from the model in each step until the minimum value of the AIC was reached. As a result, the ‘final models’ represent the models of the best fit with the lowest AIC value of each data set^94^.

Covariates that entered in the variable selection process were age (in years), sex (ref ‘female’), IgG (U/ml), ADHD (ref ‘no’), borderline personality disorder (BPD) as the most frequent Axis II disorder in this sample (ref ‘no’), Axis II other than BPD (ref ‘no’), anxiety disorder (ref ‘no’), affective disorders (ref ‘no’), lifetime substance abuse (ref ‘no’), eating disorder (ref ‘no’), antidepressant medication (ref ‘no’), antipsychotic medication (ref ‘no’), ADHD medication (ref ‘no’), and sedatives (ref ‘no’). In addition, ordinary linear regression models were estimated with IgG as a single covariate to offer minimal models for comparison.

In addition, exploratory analyses, separate linear regression models were constructed for the ADHD Index with either the *T. gondii* seropositivity (ref ‘no’) or the avidity (measured as share of affinity). For sensitivity, these models were estimated for the entire study and for the ADHD group. Linear regression analysis results were reported as regression coefficients with 95% CIs. Statistical analyses were conducted with SPSS Version 25 and R 3.5.0.

## Supplementary information

Supplementary information.
