# Supplementary material for: Aggravation of symptom severity in adult attention-deficit/hyperactivity disorder by latent Toxoplasma gondii infection: a case–control study
Source: Sci Rep. 2020 Sep 1;10:14382. doi: 10.1038/s41598-020-71084-w (PMC7463265; doi:10.1038/s41598-020-71084-w)
Supplement: Supplementary file 1 — Supplementary information. [file 41598_2020_71084_MOESM1_ESM.pdf]

# Aggravation of symptom severity in adult attention-deficit/hyperactivity disorder by latent *Toxoplasma gondii* infection - A case-control study

Alexandra P. Lam; Dominik de Sordi; Helge H.O. Müller; Martin C. Lam; Angelika Carl; Klaus P. Kohse; Alexandra Philipsen

# Supplement

**Supplementary Table 1. Diagnostic instruments.**

| | |
|---|---|
| **ADHD** | Self-rating instrument for the diagnosis of ADHD in adults ADHD Self-Rating Scale (ADHD-SR, German Version[1], covers DSM-IV criteria; Cut-off >15)<br>Wender-Utah-Rating-Scale–German short version (WURS-k; retrospective assessment of ADHD in childhood; mother rating; Cut-off >30[2]) |
| **Comorbid disorders** | Structured clinical interview for DSM-IV (SCID-I, SCID-II; covering axis-I and personality disorders; German version[3]); Self-rating instrument for specific assessment of borderline-typical symptoms (BSL-23, short-version[4]); multiple-choice self-report inventory for assessment of depression (Beck Depression Inventory (BDI-II)[5], revised version 1996); self-rating form to assess autistic symptoms (Autismus-Spektrum-Quotienten (AQ), Cut-off 30[6]) |
| **Demographics, anamnesis, risk factors:** | Additional semi-structured interview |
| **Conners adult ADHD rating scale, self-rated, long version, (CAARS-S:L)** | The self-report instrument contains 66 Items and 9 subscales. Four factor-derived scales assess ADHD-related behavior and symptoms cross-sectional (inattentive symptoms/memory problems, hyperactivity, impulsivity/emotional instability and self-concept). A 12-item ADHD Index and a Inconsistency Index are included.<br>CAARS-S:L met the criteria standards for good fit with regard to reliability, internal consistency and validity.[7-9] |
| **Specific socioeconomic status and risk-factor questionnaire** | A specific ''socioeconomic and risk factor questionnaire'' was administered capturing<br>(a) data on socioeconomic status and educational background of participants;<br>(b) date on behavioral risk factors for infection with T. gondii |

**Supplementary Table 2. Results of diagnostic instruments in ADHD patients and controls.**

| | N | Mean | SD | Minimum | Maximum |
|---|---|---|---|---|---|
| ADHD | | | | | |
| Score WURS-k | 69 | 43.0 | 14.0 | 11 | 75 |
| Score ADHS-SR | 70 | 32.0 | 8.8 | 13 | 51 |
| Score BDI-II | 70 | 17.8 | 9.6 | 1 | 41 |
| BSL-23 mean | 70 | 0.9 | 0.7 | 0.1 | 2.9 |
| Controls | | | | | |
| Score WURS-k | 70 | 10.2 | 9.0 | 0 | 56 |
| Score ADHS-SR | 70 | 5.8 | 4.8 | 0 | 19 |
| Score BDI-II | 70 | 4.4 | 3.9 | 0 | 21 |
| BSL-23 mean | 69 | 0.18 | 0.2 | 0 | 0.9 |

**Supplementary Table 3. Linear regression model of *Toxoplasma gondii* seropositivity and ADHD Index, ADHD cases only.**

| **Minimal model (n=70)** | **ADHD Index** (adjusted $R^2 = 0.060$) | | | |
|---|---|---|---|---|
| | | **95% CI** | | |
| | **B** | **Lower** | **Upper** | **p-value** |
| (Intercept) | 21.17 | 19.64 | 22.70 | **<0.001** |
| *T. gondii* (pos) | 3.41 | 0.48 | 6.34 | **0.023** |
| **Final model (n=70)** | **ADHD Index** (adjusted $R^2 = 0.163$) | | | |
| | | **95% CI** | | |
| | **B** | **Lower** | **Upper** | **p-value** |
| (Intercept) | 19.81 | 18.13 | 21.48 | **<0.001** |
| *T. gondii* (pos) | 3.60 | 0.83 | 6.37 | **0.012** |
| BPD | 3.96 | 0.86 | 7.07 | **0.013** |
| Anxiety disorder | 2.61 | -0.49 | 5.7 | 0.098 |

ADHD Index, Conners' Adult ADHD Rating Scale ADHD Index, long version; *T. gondii*, *Toxoplasma gondii*; pos, seropositive; ADHD, attention-deficit/hyperactivity disorder; BPD, borderline personality disorder; significant results in bold

**Supplementary Table 4. Stepwise regression analysis of serointensity and CAARS, all cases.**

| Minimal model (n=139) | CAARS I/EL (adjusted $R^2$ = 0.033) | | | | CAARS SKP (adjusted $R^2$ = 0.003) | | | |
|---|---|---|---|---|---|---|---|---|
| | | 95% CI | | | | 95% CI | | |
| | B | Lower | Upper | p-value | B | Lower | Upper | p-value |
| (Intercept) | 12.51 | 10.93 | 14.08 | **<0.001** | 7.69 | 6.79 | 8.59 | **<0.001** |
| IgG [U/ml] | 0.04 | 0.01 | 0.08 | **0.018** | 0.01 | -0.01 | 0.03 | 0.234 |
| **Final model (n=139)** | **CAARS I/EL** (adjusted $R^2$ = 0.696) | | | | **CAARS SKP** (adjusted $R^2$ = 0.492) | | | |
| | | **95% CI** | | | | **95% CI** | | |
| | **B** | **Lower** | **Upper** | **p-value** | **B** | **Lower** | **Upper** | **p-value** |
| (Intercept) | 5.77 | 4.58 | 6.96 | **<0.001** | 4.47 | 3.61 | 5.33 | **<0.001** |
| IgG [U/ml] | 0.02 | 0.00 | 0.04 | **0.093** | 0.00 | -0.01 | 0.02 | 0.627 |
| ADHD | 12.11 | 10.22 | 13.99 | **<0.001** | 5.07 | 3.68 | 6.47 | **<0.001** |
| BPD | 4.97 | 1.72 | 8.23 | **0.003** | 3.72 | 1.55 | 5.89 | **<0.001** |
| Gender: male | - | - | - | - | - | - | - | - |
| Anxiety disorder | 2.63 | -0.14 | 5.40 | 0.063 | 2.67 | 0.71 | 4.63 | **0.008** |
| Antidepressants | 2.58 | -0.48 | 5.64 | 0.098 | - | - | - | - |
| Hypnotics, sedatives | -5.11 | -11.39 | 1.17 | 0.110 | - | - | - | - |
| Axis II other than BPD | - | - | - | - | 2.05 | -0.65 | 4.75 | 0.135 |

CAARS, self-rated Conners' Adult ADHD Rating Scale; I/EL, impulsivity and emotional lability; SKP, problems with self-concept; IgG, immunoglobulin G; ADHD, attention-deficit/hyperactivity disorder; BPD, borderline personality disorder; significant results in bold

**Supplementary Table 5. Stepwise regression analysis of serointensity and CAARS subscales, all cases.**

| **Minimal model (n=139)** | **CAARS UA** (adjusted $R^2$ = 0.031) | | | | **UA-DSM** (adjusted $R^2$ = 0.034) | | | |
|---|---|---|---|---|---|---|---|---|
| | | **95% CI** | | | | **95% CI** | | |
| | **B** | **Lower** | **Upper** | **p-value** | **B** | **Lower** | **Upper** | **p-value** |
| (Intercept) | 13.74 | 12.23 | 15.25 | **0.000** | 10.11 | 8.78 | 11.44 | **<0.001** |
| IgG [U/ml] | 0.04 | 0.01 | 0.07 | **0.022** | 0.04 | 0.01 | 0.07 | **0.016** |
| **Final model (n=139)** | **CAARS UA** (adjusted $R^2$ = 0.683) | | | | **UA-DSM** (adjusted $R^2$ = 0.747) | | | |
| | | **95% CI** | | | | **95% CI** | | |
| | **B** | **Lower** | **Upper** | **p-value** | **B** | **Lower** | **Upper** | **p-value** |
| (Intercept) | 6.20 | 4.79 | 7.60 | **0.000** | 4.05 | 3.13 | 4.96 | **<0.001** |
| IgG [U/ml] | 0.01 | -0.01 | 0.03 | 0.352 | 0.01 | 0.00 | 0.03 | 0.120 |
| ADHD | 12.53 | 10.71 | 14.35 | **0.000** | 12.03 | 10.69 | 13.37 | **<0.001** |
| BPD | - | - | - | - | - | - | - | - |
| Gender: male | 2.17 | 0.51 | 3.82 | **0.011** | - | - | - | - |
| Anxiety disorder | 3.07 | 0.36 | 5.78 | **0.027** | 3.45 | 1.38 | 5.52 | **0.001** |
| Antidepressants | 2.10 | -0.50 | 4.70 | 0.112 | - | - | - | - |

CAARS, self-rated Conners' Adult ADHD Rating Scale; UA, inattention/memory problems; UA-DSM, inattention/memory problems according to DSM; IgG, immunoglobulin G; ADHD, attention-deficit/hyperactivity disorder; BPD, borderline personality disorder; significant results in bold

**Supplementary Table 6. Linear regression model of avidity and ADHD Index, ADHD cases only.**

| **Minimal model (n=70)** | **ADHD Index** (adjusted $R^2 = 0.062$) | | | |
|---|---|---|---|---|
| | | **95% CI** | | |
| | **B** | **Lower** | **Upper** | **p-value** |
| (Intercept) | 21.19 | 19.68 | 22.70 | **<0.001** |
| Avidity | 7.88 | 1.19 | 14.57 | **0.022** |
| **Final model (n=70)** | **ADHD Index** (adjusted $R^2 = 0.159$) | | | |
| | | **95% CI** | | |
| | **B** | **Lower** | **Upper** | **p-value** |
| (Intercept) | 19.89 | 18.23 | 21.55 | **<0.001** |
| Avidity | 7.98 | 1.65 | 14.32 | **0.014** |
| BPD | 3.72 | 0.62 | 6.83 | **0.019** |
| Anxiety disorder | 2.72 | -0.38 | 5.83 | 0.084 |

ADHD Index, Conners' Adult ADHD Rating Scale ADHD Index, long version; ADHD, attention-deficit/hyperactivity disorder; BPD, borderline personality disorder; significant results in bold

## References


1. Rosler M. Retz W, Retz-Junginger P, Thome J, Supprian T, Nissen T*, et al*. Tools for the diagnosis of attention-deficit/hyperactivity disorder in adults. Self-rating behaviour questionnaire and diagnostic checklist. *Der Nervenarzt* 2004; **75**(9)**:** 888-895.

2. Retz-Junginger P, Retz W, Blocher D, Stieglitz RD, Georg T, Supprian T*, et al*. [Reliability and validity of the Wender-Utah-Rating-Scale short form. Retrospective assessment of symptoms for attention deficit/hyperactivity disorder]. *Der Nervenarzt* 2003; **74**(11)**:** 987-993.

3. Wittchen H-U, Zaudig M, Fydrich T (1997). SKID–Strukturiertes Klinisches Interview für DSM IV. Achse I und II. . Hogrefe: Göttingen

4. Wolf M, Limberger MF, Kleindienst N, Stieglitz R-D, Domsalla M, Philipsen A*, et al*. Kurzversion der Borderline-Symptom-Liste (BSL-23): Entwicklung und Überprüfung der psychometrischen Eigenschaften. *Psychother Psych Med* 2009; **59**(08)**:** 321-324.

5. Beck A, Steer R, Brown G. *Beck Depression Inventory*. Psychological Corp: San Antonio, TX, 1996.

6. Freitag CM, Retz-Junginger P, Retz W, Seitz C, Palmason H, Meyer J*, et al*. Evaluation der deutschen Version des Autismus-Spektrum-Quotienten (AQ) - die Kurzversion AQ-k. *Zeitschrift für Klinische Psychologie und Psychotherapie* 2007; **36**(4)**:** 280-289.

7. Conners CK, Erhardt D, Sparrow EP. *Conners' adult ADHD rating scales, technical manual*. Multi-Health Systems: New York, 1999.

8. Christiansen H, Kis B, Hirsch O, Philipsen A, Henneck M, Panczuk A*, et al*. German validation of the Conners Adult ADHD Rating Scales-self-report (CAARS-S) I: factor structure and normative data. *European psychiatry : the journal of the Association of European Psychiatrists* 2011; **26**(2)**:** 100-107.

9. Christiansen H, Hirsch O, Philipsen A, Oades RD, Matthies S, Hebebrand J*, et al*. German validation of the conners adult ADHD rating scale-self-report: confirmation of factor structure in a large sample of participants with ADHD. *Journal of attention disorders* 2013; **17**(8)**:** 690-698.

10. Spielberger CD, Schaffner P, Laux P (1983). State-Trait-Angstinventar (STAI). BELTZTEST.

11. Schwenkmezger P, Hodapp V, Spielberger C (1992). State-Trait-Ärgerausdrucks-Inventar. Hans Huber.
